# Hippocampal morphological atrophy and distinct patterns of structural covariance network in Alzheimer’s disease and mild cognitive impairment

**DOI:** 10.3389/fpsyg.2022.980954

**Published:** 2022-09-09

**Authors:** Dawei Miao, Xiaoguang Zhou, Xiaoyuan Wu, Chengdong Chen, Le Tian

**Affiliations:** ^1^School of Automation, Beijing University of Posts and Telecommunications, Beijing, China; ^2^School of Economics and Management, Minjiang University, Fuzhou, China; ^3^School of Electrical and Information Engineering, Beijing University of Civil Engineering and Architecture, Beijing, China

**Keywords:** Alzheimer’s disease, mild cognitive impairment, gray matter volume, structural covariance, distinct patterns

## Abstract

Elucidating distinct morphological atrophy patterns of Alzheimer’s disease (AD) and its prodromal stage, namely, mild cognitive impairment (MCI) helps to improve early diagnosis and medical intervention of AD. On that account, we aimed to obtain distinct patterns of voxel-wise morphological atrophy and its further perturbation on structural covariance network in AD and MCI compared with healthy controls (HCs). T1-weighted anatomical images of matched AD, MCI, and HCs were included in this study. Gray matter volume was obtained using voxel-based morphometry and compared among three groups. In addition, structural covariance network of identified brain regions exhibiting morphological difference was constructed and compared between pairs of three groups. Thus, patients with AD have a reduced hippocampal volume and an increased rate of atrophy compared with MCI and HCs. MCI exhibited a decreased trend in bilateral hippocampal volume compared with HCs and the accelerated right hippocampal atrophy rate than HCs. In AD, the hippocampus further exhibited increased structural covariance connected to reward related brain regions, including the anterior cingulate cortex, the putamen, the caudate, and the insula compared with HCs. In addition, the patients with AD exhibited increased structural covariance of left hippocampus with the bilateral insula, the inferior frontal gyrus, the superior temporal gyrus, and the cerebellum than MCI. These results reveal distinct patterns of morphological atrophy in AD and MCI, providing new insights into pathology of AD.

## Introduction

Alzheimer’s disease (AD) is an irreversible neurodegenerative process resulting into severe mental dysfunctions and ultimately causes death (Hett et al., 2021). The feature of AD is the accumulation of beta-amyloid plaques and neurofibrillary tangles, resulting into progressive synapse and neuronal losses (Hardy, 2006). What is worse, there is no therapy that can stop or hinder this progression. Since AD develops much earlier than the clinical phenotypic manifestation, identifying predictors at its early stage really matters in AD research (Talwar and Kushwaha, 2021). Mild cognitive impairment (MCI) draws much attention as a preclinical transitional state between healthy subjects and AD (Petersen et al., 2001), with the annual progression rate from MCI to AD 5–27% (Liu et al., 2010). Investigating neuroimaging phenotype between AD and MCI helps with early diagnosis and medical intervention of MCI before it converts to AD.

Neuroimaging studies have identified morphological atrophy in AD and MCI. For example, decreased hippocampal and entorhinal cortex volumes are found in MCI and AD (Devanand et al., 2007). In addition, the hippocampal atrophy rate and the whole brain atrophy rate can predict the progression to AD from MCI (Henneman et al., 2009). Volumes of distributed brain regions, including the frontal gyrus, the temporal gyrus, and even the whole brain, are found to be potential biomarkers of conversion from MCI to AD (Liu et al., 2010). However, these studies focus on predefined region-of-interest (ROI) volume and are limited by methodological differences, such as the arbitrary delineation of the ROI and heterogeneity of manual morphometry by human raters (Ashburner and Friston, 2000). In recent years, voxel-based morphometry (VBM) has been proposed to overcome these limitations (Ashburner and Friston, 2000). As an objective and automated method without manual morphometry, VBM shows numbers of advantages over manual morphometry (Ashburner and Friston, 2000). On the other hand, there is a trending to investigate the pathology of AD at the network level other than focusing on specific brain areas (Alexander-Bloch et al., 2013). Brain regions subserving particular behavioral or cognitive functions exhibit coordinated volume, termed as structural covariance (Evans, 2013). Structural covariance between regions is hypothesized to originate from genetic influence on development (Evans, 2013) and can be influenced by mutual brain-derived neurotrophic factor (Pezawas et al., 2004) and activity-dependent structural plasticity (Draganski et al., 2004). Comparing with widely used functional connectivity, structural covariance measures more stable and trait-like brain connectivity (Evans, 2013). However, how local volume atrophy identified using VBM perturbs its large-scale structural covariance in AD and MCI remains unknown.

In this study, matched AD (*n* = 38), MCI (*n* = 87), and HCs (*n* = 29) were downloaded from the Alzheimer’s Disease Neuroimaging Initiative (ADNI) open database.^1^ Our aims were twofold. First, we investigated voxel-wise gray volume differences among AD, MCI, and HCs where voxel-wise gray volume was obtained using VBM. As morphological atrophy was consistently found in distributed brain regions in previous studies, we expected to find decreased gray matter volume in AD and MCI. Then, identified regions were chosen as seeds to construct structural covariance network. We investigated the differential structural covariance network of identified regions among three groups.

## Materials and methods

### Subjects

All the subjects used in this study come from the Alzheimer’s Disease Neuroimaging Initiative (ADNI) open database (see footnote 1). Since 2013, this project has recruited over 1,500 adults (55–90 years old), consisting of patients with AD, MCI subjects, and healthy controls. All the subjects used in this study come from ADNI Phase 2 (ADNI 2). Thirty-eight patients with AD, 87 patients with MCI, and matched healthy controls (HCs) were included in this study. Cognitive function and degree of dementia were evaluated by the Mini-Mental State Examination (MMSE) and Clinical Dementia Rating Scale-Sum of Boxes (CDR_SB) (Folstein et al., 1975; O’Bryant et al., 2008). More details are included in Table 1. The exclusion criteria of all the subjects were: 1) comorbid with depression; 2) having other kind of dementia.

**TABLE 1:** Demographic and clinical information of participants.

	AD (*n* = 38)	MCI (*n* = 87)	HC (*n* = 29)	*p*
Sex (female/male)	14/24	23/64	11/18	0.35^a^
Age (mean ± SE)	72.68 ± 7.32	72.11 ± 7.14	73.93 ± 5.62	0.47^b^
MMSE (mean ± SE)	20.65 ± 3.55	26.23 ± 3.51	28.93 ± 1.02	<0.001^b^
CDR_SB (mean ± SE)	4.17 ± 1.68	1.64 ± 2.81	0.00 ± 0.00	<0.001^b^

^a^Chi-square t-test. ^b^One-way ANOVA. MMSE, Mini-Mental State Examination; CDR_SB, Clinical Dementia Rating Scale-Sum of Boxes.

### Data acquisition

T1-weighted anatomical images were acquired on a 3.0 T Philips Healthcare MRI scanner, with the parameters as follows: a flip angle = 9 degrees, matrix = 256 × 256 × 170, slice thickness = 1.2 mm, TR = 6.67 ms, TE = 3.1 ms, pixel spacing = 1 mm× 1 mm× 1.2 mm^3^.

### Voxel based morphometry analysis

Voxel based morphometry (VBM) was adopted to obtain gray matter volumes following the standard pipeline of CAT 12 toolbox.^2^ The details referred to (Ashburner, 2009). Main steps included bias-field correction, segmentation (into gray and white matter and cerebrospinal fluid), adjustment for partial volume effects, normalization into Montreal Neurological Institute space, resampled to 1.5 mm × 1.5 mm × 1.5 mm and nonlinear modulation (Ashburner, 2009). Finally, the gray matter maps were smoothed using 6-mm full width at half maximum Gaussian kernel (FWHM). As nonlinear modulation was adopted, we did not include the total brain volume as an additional covariate in the following statistical analysis (Bludau et al., 2016).

### Statistical analysis

One-way ANOVA was done to determine differential voxel-wise gray matter volume across three groups. This procedure was done in SPM 12 with age and sex as covariates. Results were corrected for multiple comparison with Gaussian random filed (GRF) (voxel-wise *p* < .005, cluster-level *p* < .05). Then, the mean GMVs of each peak coordinate with the spherical radius of 6 mm were extracted and compared between each pair of groups with two sample *t*-tests.

### Structural covariance analysis

Then, we investigated whether structural covariance network of identified regions was also abnormal in patients (AD/MCI). Each peak coordinate of GMV aberrance (ANOVA results) with a spherical radius of 6 mm was defined as seed; structural covariance of the seed was constructed and compared between each pair of three groups. The model was defined as follows:


$$V_{i}=\beta_{0}+\beta_{1}*S\,e\,x+\beta_{2}*A\,g\,e+\beta_{3}*V_{s\,e\,e\,d}$$

where *V*_*i*_was the gray matter volume of voxel i,*V_seed_* was the mean gray matter volume of one seed. Age and sex were included as covariates. The β_3_ was compared between each pair of three groups using two sample *t*-tests. Results were corrected for multiple comparisons (voxel-wise *p* < .005, cluster-level *p* < .05, GRF corrected). This comparison was done between pairs of three groups (AD vs. MCI, MCI vs. HCs, and AD vs. MCI).

## Results

### Voxel-wise gray matter volume analysis results

**FIGURE 1:**
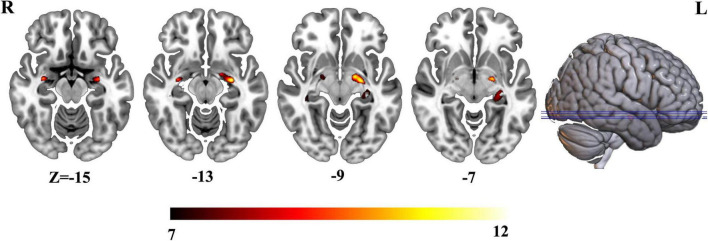
Differential voxel-wise gray matter volume across three groups.

**FIGURE 2:**
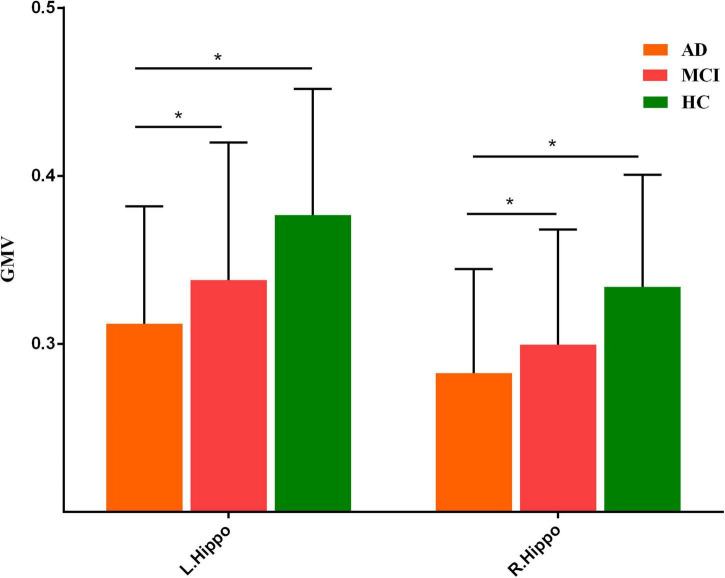
*Post hoc* results of gray matter volume difference in bilateral hippocampus. “*” meant *p* value < 0.05.

Three groups exhibited significant gray matter volume difference in bilateral hippocampus (Figure 1 and Supplementary Table 1). The *post hoc* results showed that the patients with AD exhibited decreased bilateral hippocampal volume than MCI and HCs. MCI exhibited a decreased trend in bilateral hippocampal volume compared with HCs (Figure 2).

**FIGURE 3:**
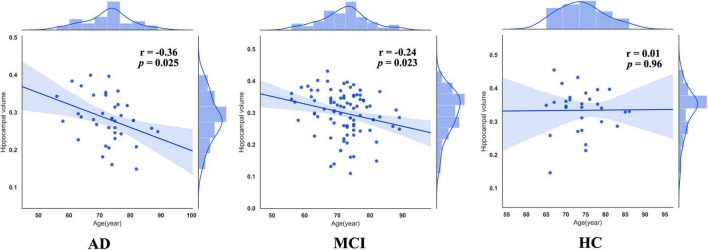
Correlations between right hippocampus in three groups.

As previous studies have pointed out, patients with AD were accompanied by accelerated atrophy of hippocampal volume comrade with normal aging. Thus, we extracted mean GMVs of each peak coordinate (bilateral hippocampus) with a spherical radius of 6 mm and calculated the correlation between them and age. This procedure was done in each group, respectively. As a result, right hippocampal volumes in patients with AD (*r* = –0.36, *p* = 0.025) and MCI (*r* = –0.24, *p* = 0.023) were negatively correlated with age, while those in HCs (*r* = 0.01, *p* = 0.96) were not (Figure 3).

### Structural covariance analysis results

**FIGURE 4:**
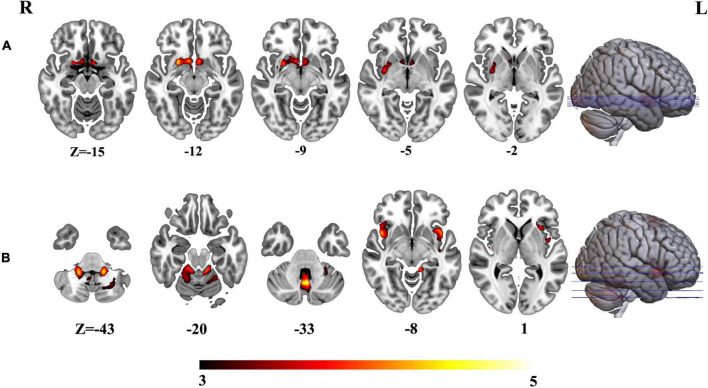
**(A)** Altered structural covariance of left hippocampus in AD compared with HCs. **(B)** Altered structural covariance of left hippocampus in AD compared with MCI.

To further investigate the altered structural covariance network of identified regions whose volume was altered among three groups, we constructed structural covariance of bilateral hippocampus and compared between pairs of three groups (AD vs. MCI, MCI vs. HCs and AD vs. MCI). As a result, the patients with AD demonstrated significantly increased structural covariance of bilateral hippocampus compared with HCs. As bilateral hippocampus exhibited similar aberrance patterns, we just reported altered structural covariance of left hippocampus in the main text. The altered structural covariance of right hippocampus was included in Supplementary Figure 1. The patients with AD exhibited increased structural covariance of left hippocampus connected with brain regions, including the insula, the putamen, the caudate, the anterior cingulate cortex (ACC)/ventral medial prefrontal cortex (vmPFC) (Figure 4A and Supplementary Table 2). There was no significant difference between MCI and HCs in terms of structural covariance network of bilateral hippocampus. Compared with MCI, the patients with AD exhibited increased structural covariance connected to the bilateral insula, the inferior frontal gyrus, the superior temporal gyrus, and the cerebellum (Figure 4B and Supplementary Table 3).

## Discussion

In this study, we investigated voxel-wise gray matter volume atrophy and its perturbation on structural covariance network of AD and MCI compared with HCs. The patients with AD exhibited decreased hippocampal volume and an increased atrophy rate compared with MCI and HCs. MCI exhibited a decreased trend in bilateral hippocampal volume and accelerated hippocampal atrophy compared with HCs. Left hippocampus exhibited increased structural covariance connected to reward related brain regions in AD compared with HCs, including the ACC/vmPFC, the putamen, the caudate, and the insula compared with HCs. In addition, the patients with AD exhibited increased structural covariance of left hippocampus with the bilateral insula, the inferior frontal gyrus, the superior temporal gyrus, and the cerebellum than MCI.

Structural atrophy and dysfunction of hippocampus were consistently reported in AD. Playing an important role in declarative memory, the hippocampus was identified as the anatomical signature of AD (Schröder and Pantel, 2016). Both AD and MCI were found to be associated with hippocampal atrophy and the faster atrophy rate (Machado et al., 2020), even before clinical symptoms occurred (Scahill et al., 2002; Schott et al., 2003). Hippocampal atrophy, especially its atrophy rate, turned out to be a potential biomarker to predict the conversion from MCI to AD (Henneman et al., 2009). Studies using longitudinal data found that hippocampal volume predicted conversion from MCI to AD after 2 years (Karas et al., 2004). During the progression of AD, hippocampal atrophy (rates) (Chan et al., 2003) was a consequence of dysfunction of neurotrophin signaling complexes (Ginsberg et al., 2019). From this point of view, AD was expected to have smaller hippocampal volume than MCI and HCs. Actually, studies found that atrophy (rates) of hippocampus distinguished AD, MCI, and HCs (Mungas et al., 2002; Karas et al., 2004; Chen et al., 2021). However, other studies found that hippocampal atrophy (rate) could not differentiate patients with AD from patients with MCI (Jack et al., 2000; Henneman et al., 2009). The reasons were manifold, such as methodologies, sample, and scan protocols. Consistent with these studies, we observed decreased hippocampal atrophy in AD compared with HCs and MCI. MCI also exhibited a decreasing trend in hippocampal atrophy compared with HCs. Correlation results further revealed more accelerated hippocampal atrophy in AD than MCI.

In addition, we also observed increased structural covariance of hippocampus in AD compared with HCs. In addition to its regional aberrance, the hippocampus was also implanted with large-scale brain networks. Structural covariance measured brain connectivity through coordinated interregional volumes (Alexander-Bloch et al., 2013). There was a trend to investigate degenerative diseases on brain networks other than individual regions. Altered interregional structural covariance patterns were already found in neurodegenerative diseases, including AD, revealing the pathology of these diseases at the network level (Alexander-Bloch et al., 2013). In this study, we identified altered structural covariance network of hippocampus in AD compared with HCs in reward-related brain regions, including ACC, the putamen, the caudate, and the insula. Dysfunction of these regions also played important roles in the pathology of AD. For example, the presence of amyloid deposition in the striatum was found at the beginning of AD (Braak and Braak, 1990; Klunk et al., 2007). The smaller volume of striatum was associated with worse executive function in AD (Wolf et al., 2004; Roh et al., 2011). Combined with previous findings, the increased structural covariance between key regions of the reward system and hippocampus might be a result from correlated gray matter loss of these regions by the same neurodegenerative process (Alexander-Bloch et al., 2013). Altered structural covariance between hippocampus and key regions in reward system might be related to apathy in AD. As the most common neuropsychiatric symptom in AD (Zhao et al., 2016), apathy was related to diminished motivation (Rosenberg et al., 2015). Neuroimaging studies identified that the apathy was underlain by dysfunction of rewards system, especially for ACC (Rosen et al., 2005). Correlation between hypoperfusion of the ACC and apathy was found in AD (Lanctôt et al., 2007). In addition to ACC, the frontostriatal circuit along with parts of basal ganglia was also hypothesized to a play pivotal role in apathy across neurodegenerative disorders (Theleritis et al., 2014; Le Heron et al., 2018). Taken together, our results suggested that gray matter loss of reward system was coordinated other than isolated and might be related to apathy in AD.

The increased structural covariance of hippocampus connected to regions, including the insula, the frontal gyrus, the temporal gyrus, and the cerebellum, was also identified in this study. As we mentioned earlier, hippocampal atrophy (rate) could distinguish patients with AD from MCI (Mungas et al., 2002; Karas et al., 2004; Ferreira et al., 2011). The increased structural covariance of these regions connected to hippocampus hinted the atrophy rate of these regions was increased in AD compared with MCI. When progressed to AD, MCI was accompanied by greater gray matter loss in multiple brain regions, including the hippocampus, the inferior and middle temporal gyri (Chételat et al., 2005; Spulber et al., 2012). A longitudinal study showed that MCI who converted to AD exhibited reduced volumes of gray matter in the insula and the temporal and frontal gyri compared with MCI who did not convert to AD (Davatzikos et al., 2011). Consistent with these studies, our results suggested that gray matter volume atrophy in these brain regions played important roles in transition from MCI to AD.

There were several limitations in this study. First, according to the longitudinal change, MCI was further divided into two subgroups, namely, stable and progressive MCI (Davatzikos et al., 2011; Spulber et al., 2012). Significant difference was observed in these two subtypes in gray matter volume. We did not consider subtypes in this study. Second, our results were obtained based on one single dataset; future studies could use an independent dataset to validate these results. Third, functional aberrance was also observed in AD and MCI; the relationship between structural and functional aberrance could be investigated in future studies.

## Conclusion

In this study, we identified hippocampal atrophy (rate) and its perturbation on structural covariance network in AD compared with HCs and MCI. AD exhibited increased structural covariance network of hippocampus connected to reward-related brain regions than HC, hinting coordinated atrophy of these regions. In addition, compared with MCI, AD exhibited increased structural covariance between the left hippocampus and the bilateral insula, the inferior frontal gyrus, the superior topic gyrus, and the cerebellum, suggesting that gray matter volume atrophy in these brain regions plays an important role in the transition from MCI to AD.

## Data availability statement

Publicly available datasets were analyzed in this study. This data can be found here: https://ida.loni.usc.edu/login.jsp.

## Ethics statement

Ethical review and approval was not required for the current study in accordance with the local legislation and institutional requirements. The datasets on which this article relies were reviewed and approved by Cleveland Clinic Institutional Review Board ADNI Individual Site Institutional Review Board. Written informed consent for participation was not required for this study in accordance with the national legislation and the institutional requirements.

## Author contributions

DM designed the research, analyzed the data, and wrote the manuscript. XW analyzed the data and wrote the manuscript. CC searched the literature and downloaded the data. LT provided suggestions and modified the language. XZ directed the research program and provided guidance and suggestions for the study. All authors read and approved the final manuscript.

## Footnotes

1
http://adni.loni.usc.edu/

2
http://dbm.neuro.uni-jena.de/cat12/
