# Supplementary material for: Hippocampal morphological atrophy and distinct patterns of structural covariance network in Alzheimer’s disease and mild cognitive impairment
Source: Front Psychol. 2022 Sep 9;13:980954. doi: 10.3389/fpsyg.2022.980954 (PMC9505506; doi:10.3389/fpsyg.2022.980954)

**Table S1**. Voxel-wise gray matter volume difference among three groups.

| Cluster | No. of voxels | Peak coordinate | Including regions | F |
| --- | --- | --- | --- | --- |
| 1 | 748 | -29,-11,-12 | Left hippocampus | 12.28 |
|  |  |  | Parahippocampa Gyrus |  |
| 2 | 411 | 32,-9,-14 | Right hippocampus | 10.23 |
|  |  |  | Parahippocampa Gyrus |  |

**Table S2**. Altered structural covariance of left hippocampus in AD compared with HCs.

| Cluster | No. of voxels | Peak coordinate | Including regions | T |
| --- | --- | --- | --- | --- |
| 1 | 1901 | 23,9,-12 | Right putamen | 4.45 |
|  |  |  | Right caudate |  |
|  |  |  | Anterior Cingulate cortex |  |

**Table S3**. Altered structural covariance of left hippocampus in AD compared with MCI.

| Cluster | No. of voxels | Peak coordinate | Including regions | T |
| --- | --- | --- | --- | --- |
| 1 | 8715 | -3,-62,-33 | Cerebellum Anterior Lobe | 4.8311 |
|  |  |  | Cerebellum Posterior Lobe |  |
| 2 | 2107 | -39,15,-5 | Left Insula | 4.3542 |
|  |  |  | Left Inferior Frontal Gyrus |  |
|  |  |  | Left Superior Temporal Gyrus |  |
| 3 | 1734 | 44,11,-6 | Right Insula | 4.5141 |
|  |  |  | Right Inferior Frontal Gyrus |  |
|  |  |  | Right Superior Temporal Gyrus |  |
| 4 | 1491 | 12,-9,68 | Right Superior Frontal Gyrus | 3.9201 |
|  |  |  | Right Medial Frontal Gyrus |  |

**Figure S1**. Altered structural covariance of right hippocampus in AD compared with HCs.


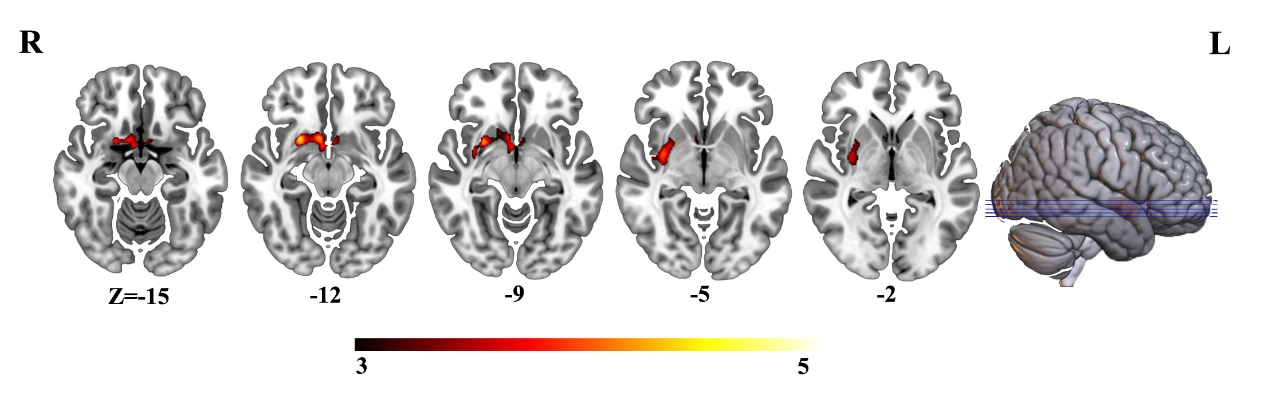

Supplement: Supplementary file 1 [file Data_Sheet_1.docx]
